# Infectious Burden and Immortal-Time Bias During AML Induction: A Supportive-Care Cohort Under Posaconazole Prophylaxis

**DOI:** 10.3390/jcm15207841

**Published:** 2026-10-11

**Authors:** Hilmi Erdem Gözden, Halime Betül Tokat, Hamide Tuğba Uluçay, Fatma Tuğçe Bayrak, Recep Kaya, Alperen Burak Kocabıyık, Süleyman Emre Okutan, İbrahim Ethem Pınar, Erdal Kurtoğlu, Volkan Karakuş

**Affiliations:** 1 Department of Hematology, Bursa Yüksek İhtisas Training and Research Hospital, University of Health Sciences, 16310 Bursa, Turkey; 2 Department of Internal Medicine, Antalya Training and Research Hospital, 07100 Antalya, Turkey; dr.hbtokat@gmail.com (H.B.T.); htugbap@gmail.com (H.T.U.); dr.ftbayrak@gmail.com (F.T.B.); drrecepkaya@gmail.com (R.K.); a.burakkocabiyik07@gmail.com (A.B.K.); drokutanemre@gmail.com (S.E.O.); 3 Department of Hematology, Faculty of Medicine, Bursa Uludağ University, 16059 Bursa, Turkey; iethempinar@uludag.edu.tr; 4 Department of Hematology, Antalya Training and Research Hospital, 07100 Antalya, Turkey; erdalkurtoglu@yahoo.com

**Keywords:** acute myeloid leukaemia, supportive care, infection, febrile neutropenia, early mortality, immortal-time bias, real-world evidence

## Abstract

**Background/Objectives:** Infection is a major complication of acute myeloid leukaemia (AML) induction, and analyses relating treatment-emergent infection to survival are prone to immortal-time bias. We characterised baseline and treatment-emergent infection during induction and used bias-aware methods to distinguish a pre-treatment risk signal from a survival-time artefact. **Methods:** Our study analysed a retrospective single-centre cohort of 91 adults with newly diagnosed AML receiving induction under universal posaconazole prophylaxis (2019–2025) with Cox models, 14- and 30-day landmark analyses and Firth penalised logistic regression. **Results:** Active infection at diagnosis occurred in 23/91 (25.3%) and treatment-emergent infection in 54/91 (59.3%). A naive Cox model showed an implausibly protective association for treatment-emergent infection (hazard ratio [HR] 0.46, 95% CI 0.21–0.99). In 14- and 30-day landmark analyses (HR 0.59 and 0.60; 95% CIs included 1) the estimate moved toward the null and lost statistical significance; because infection-onset dates were unavailable, landmark analysis only partially corrects this bias, and the independent association between treatment-emergent infection and survival could not be reliably estimated. Active infection at diagnosis showed a fragile, exploratory association with later mortality that was nominally significant in only one of several pre-specified specifications (30-day landmark HR 2.56, 95% CI 1.03–6.37). **Conclusions:** The apparently protective association of treatment-emergent infection with survival is consistent with immortal-time bias; it is a complication and marker of course, not a survival determinant. Baseline infection is a hypothesis-generating candidate pre-treatment risk marker that requires prospective validation.

## 1. Introduction

Acute myeloid leukaemia (AML) is the most common form of acute leukaemia in adults and carries among the poorest survival of the haematological malignancies, with a median age at diagnosis near 68 years and a 5-year survival of roughly one-quarter [1]. Remission-induction chemotherapy is the gateway to durable remission for eligible patients, but the early induction period is dominated by the competing risk of treatment-related death, of which infection is the leading cause [2,3]. Profound and prolonged neutropenia, mucosal-barrier injury and broad-spectrum antimicrobial exposure together create a window of intense vulnerability to bacterial, fungal and viral complications, and septic shock is the single most frequent proximate cause of death during induction [3]. Early death remains a major mode of induction failure; in real-world AML cohorts, delayed treatment initiation, multidrug-resistant sepsis, performance status and other patient-fitness factors are consistently among its strongest correlates [3,4]. Infection prevention and management (antimicrobial prophylaxis, febrile-neutropenia pathways and stewardship) is therefore a central pillar of AML supportive care [5,6].

Infectious burden in AML has two clinically distinct components that are rarely examined together. The first is infection already present at the moment of diagnosis (before any cytotoxic therapy), which may index higher disease burden, frailty or early barrier dysfunction and is, importantly, measurable before treatment begins. The second is infection emerging during induction as a treatment-associated complication. In contemporary cohorts managed with antifungal but not routine antibacterial prophylaxis, febrile episodes affect almost all patients, microbiologically documented infection roughly one-quarter, and infection-related 30-day mortality approximately 5% [3]. Bloodstream infection is dominated by Gram-negative organisms and carries a 30-day mortality of 25–30%, rising to 50–60% when the organism is carbapenem-resistant [7,8], a pressure that is especially pronounced in the Eastern Mediterranean, where multidrug-resistant (MDR) Gram-negative bacteraemia in haematology patients is endemic [8,9,10,11]. Empirical-therapy strategy is consequently guided by local resistance epidemiology [5,6,9].

The contemporary treatment landscape complicates any comparison across induction strategies. Intensive “3+7” induction, hypomethylating-agent–venetoclax (HMA–venetoclax) regimens, APL-directed therapy and reduced-intensity approaches differ in myelosuppressive profile and, critically, in the patients to whom they are offered: older and less fit patients are preferentially allocated to lower-intensity regimens [2,12,13]. Real-world series of HMA–venetoclax confirm a substantial infectious burden reflecting the frail population treated rather than a uniquely infectious regimen [13,14,15]. Universal mould-active prophylaxis with posaconazole is standard during induction at many centres [16], and invasive fungal infection (IFI) is classified by the EORTC/MSGERC 2020 consensus criteria [17].

A specific methodological hazard attends any analysis that relates treatment-emergent infection to mortality. Because a treatment-emergent infection can only be recorded in a patient who survives long enough to develop and have it documented, patients who die very early are systematically classified as “infection-free”. This is a well-recognised source of immortal-time bias, first described in the heart-transplant survival literature and repeatedly shown to generate spurious (even inverted) associations in observational studies [18,19]. Naive fixed-exposure survival models are therefore liable to conclude, implausibly, that infection improves survival, and bias-aware methods such as landmark analysis are required [18,20].

We therefore examined baseline and treatment-emergent infectious burden in a real-world AML induction cohort managed under universal posaconazole prophylaxis. We hypothesised that baseline infection and treatment-emergent infection represent distinct supportive-care constructs: the former a candidate pre-treatment risk marker, and the latter a time-dependent complication that cannot be interpreted using naive fixed-exposure survival models. Accordingly, we (i) described the infection burden at diagnosis and during induction, (ii) examined how infection frequency and mortality relate to induction intensity, and (iii) explicitly quantified and mitigated immortal-time bias when relating treatment-emergent infection to mortality. The study is retained as a descriptive endpoint only and association-focused; no causal claim is intended.

## 2. Materials and Methods

### 2.1. Study Design, Setting and Participants

**Figure 1:**
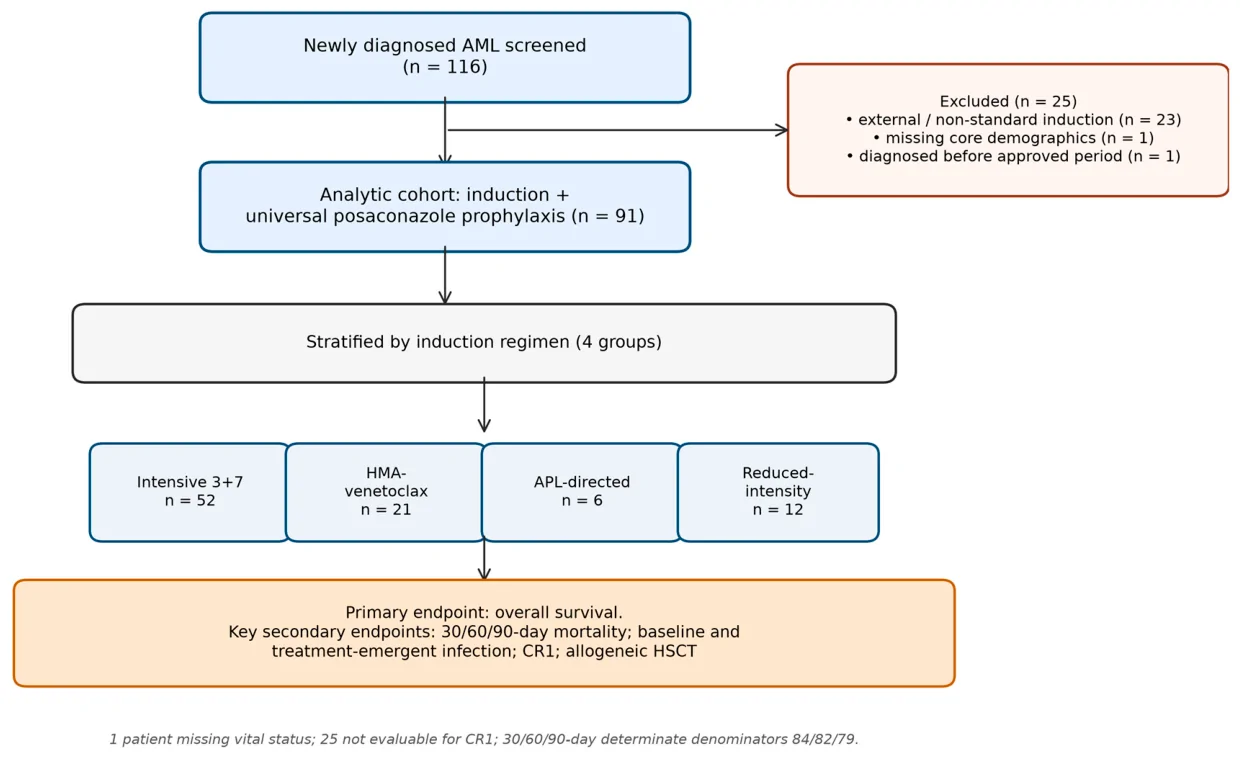
Study flow diagram. Primary endpoint: overall survival. Key secondary endpoints: 30-, 60- and 90-day mortality; baseline and treatment-emergent infection; first complete remission; and allogeneic haematopoietic stem-cell transplantation. Of the 91 included patients, 90 had known vital status (one missing) and 66 were evaluable for first complete remission; the determinate denominators for 30-, 60- and 90-day mortality were 84, 82 and 79, respectively, because patients alive but censored before each cut-off were excluded (Supplementary Table S4).

This is a retrospective single-centre observational cohort study at the Department of Haematology, Antalya Training and Research Hospital, Antalya, Turkey, reported per the STROBE statement [21]. Eligible patients were adults (≥18 years) newly diagnosed with AML (any subtype) who received remission-induction chemotherapy under universal posaconazole prophylaxis [16] between 2019 and 2025 (the period covered by the ethics-approved protocol) with documented follow-up. Of 116 patients screened, 91 met inclusion criteria (Figure 1; 23 excluded for external/non-standard induction, 1 for missing core demographics, and 1 diagnosed before the start of the ethics-approved study period).

### 2.2. Exposures

**Baseline infectious status**: active clinical infection at diagnosis, infection focus, microbiological growth at diagnosis, organism class, empiric antibiotic exposure before induction and antibiotic days. **Treatment-emergent infection**: any infection after induction start, focus, microbiological documentation, organism class, and the binary sub-types pulmonary, bloodstream, urinary and skin/soft-tissue/perianal/catheter infection. Induction regimen was mapped to four groups (intensive 3+7; HMA–venetoclax; APL-directed; reduced-intensity) and dichotomised as intensive 3+7 versus non-intensive for modelling. Operational definitions of every infectious endpoint are provided in Supplementary Table S2 and summarised in Section 2.5.

### 2.3. Endpoint Hierarchy

Because follow-up duration was heterogeneous, all-cause mortality at last follow-up was retained as a descriptive endpoint only. The primary outcome was overall survival (OS) from AML diagnosis, analysed by Cox regression and time-aware methods. Diagnosis was chosen as time zero because it is the earliest unambiguous and uniformly recorded date for every patient, and because deaths occurring between diagnosis and the start of induction (median interval 4 days, IQR 2–11, which differed across regimen groups) are themselves part of the early-mortality burden of interest. Because treatment-emergent infection can only arise after induction begins, all survival models were additionally re-anchored at induction start as a sensitivity analysis (Section 2.6; Supplementary Table S7). Key secondary outcomes were 30-, 60- and 90-day mortality (binary; see Section 2.6 for handling of patients censored alive before each landmark); treatment-emergent infection; active infection at diagnosis; breakthrough IFI (EORTC/MSGERC 2020) [17]; first complete remission (CR1; European LeukemiaNet [ELN] 2022 criteria) [2]; and allogeneic haematopoietic stem-cell transplantation (HSCT).

### 2.4. Covariates and Data Availability

Available covariates were age, sex, AML subtype (de novo/secondary/therapy-related) and diagnosis-to-treatment interval. We pre-specified an honest data-availability audit. The following were entirely absent from the source records and could not be analysed: baseline white-cell, neutrophil and platelet counts, C-reactive protein and procalcitonin (0/91); ELN 2022 genetic-risk category (not derivable: *NPM1* documented in 4/91, *TP53* in 0/91, *FLT3* tested in 24/91); ECOG performance status and comorbidity/frailty indices; neutropenia duration, depth, ANC nadir and recovery date; posaconazole trough concentrations; and infection-onset dates (Supplementary Table S1). *FLT3* status (tested 24/91, positive 11) and APL status (10/91) are reported descriptively only and were not used as model covariates.

### 2.5. Infection Definitions and Adjudication

Infectious endpoints were abstracted from clinical records using pre-specified operational criteria (full table in Supplementary Table S2). **Active infection at diagnosis**: a clinically identified infectious focus and/or documented infection present before induction start; isolated fever without a clinical, radiological or microbiological focus was not classified as active infection unless the treating team documented and treated it as a clinical infection. **Treatment-emergent infection**: a new clinically, microbiologically and/or radiologically documented infection after induction start; febrile neutropenia alone was not sufficient for classification as treatment-emergent infection unless accompanied by a documented clinical focus, microbiological evidence or radiological evidence. **Clinically documented infection**: a clinical/radiological focus without microbiological confirmation. **Microbiologically documented infection**: a clinically meaningful pathogen from blood, urine, respiratory, catheter, wound, stool or sterile-site culture; isolated coagulase-negative staphylococci were treated as contaminants unless recovered from multiple sets with a compatible syndrome, and *Candida* from non-sterile sites and asymptomatic candiduria were not counted as invasive infection. **Bloodstream infection**: a positive blood culture with a compatible systemic syndrome, applying the same skin-contaminant rule. **Pulmonary infection**: a compatible clinical syndrome supported by chest imaging (computed tomography where available, otherwise chest radiography); pulmonary events meeting EORTC/MSGERC criteria for possible/probable fungal pneumonia were additionally counted under breakthrough IFI, so a small number of pulmonary events appear in both categories and this overlap is noted where relevant. ***C. difficile* infection**: diarrhoea with a positive toxin/PCR assay. **MDR Gram-negative infection**: resistance defined per standard international categories (extended-spectrum β-lactamase [ESBL]/third-generation-cephalosporin-resistant, carbapenem-resistant). **Breakthrough IFI**: EORTC/MSGERC 2020 proven/probable/possible, reported here as a descriptive frequency among the infection endpoints. Infection adjudication was performed by treating-record review without an independent infectious-disease committee, a limitation acknowledged in Section 4.9.

### 2.6. Statistical Analysis

Continuous variables are summarised as median (IQR) and compared by Kruskal–Wallis test, and categorical variables are summarised as n (%) and compared by χ^2^ or Fisher’s exact test. Proportions are reported with Wilson 95% confidence intervals and absolute risks accompany every model estimate.

**Survival.** The primary OS model was a parsimonious Cox proportional-hazards model [22] (age + baseline infection + induction intensity), kept deliberately simple given only 29 deaths. Treatment-emergent infection was examined as an explicitly biased fixed baseline covariate and then with bias-aware methods. As a further sensitivity analysis, all Cox models were re-fitted with time zero at induction start, defining follow-up as time from diagnosis minus the recorded diagnosis-to-treatment interval (available for 86 of 90 patients; two patients without follow-up beyond induction start were excluded, leaving n = 84 with 26 deaths), with the 14- and 30-day landmarks applied from induction start (Supplementary Table S7).

**Immortal-time bias.** Because infection-onset dates were unavailable, treatment-emergent infection could not be modelled as a true time-dependent covariate; a time-dependent Cox model was therefore not estimable and is not presented. As a bias-reduction strategy we used 14- and 30-day landmark analyses [20] (restricting to survivors beyond the landmark and restarting the clock) and a sensitivity analysis excluding deaths within 30 days. The purpose of these analyses was not to estimate a definitive effect of infection, but to test whether the apparently protective naive association was robust to bias-reduction. Because the infection flag reflects whole-induction-course documentation rather than dated events, infection status before each landmark could not be defined with certainty; the landmark analyses therefore reduce, but do not eliminate, immortal-time/future-information bias, and a time-dependent or multi-state model would have been preferable had onset dates been available [19].

**Early mortality and infection.** We modelled 30-, 60- and 90-day mortality and treatment-emergent infection with Firth penalised logistic regression [23,24] using the R package logistf (version 1.26.1), which removes first-order small-sample bias and yields finite estimates under sparse-data separation. For early-mortality endpoints, patients whose last documented contact occurred before the relevant time point and who were alive at that contact were considered indeterminate rather than event-free, and were excluded; this yielded determinate denominators of 84, 82 and 79 at days 30, 60 and 90 (6, 8 and 11 patients excluded, respectively; one further patient had missing vital status). The excluded patients reflect incomplete follow-up records and transfers rather than known survival and were balanced across infection groups (Fisher *p* = 0.69 at day 30) and across induction intensity (Supplementary Table S4), and, because early censoring could underestimate early mortality, these endpoints are interpreted as descriptive and treated cautiously.

**Figure 2:**
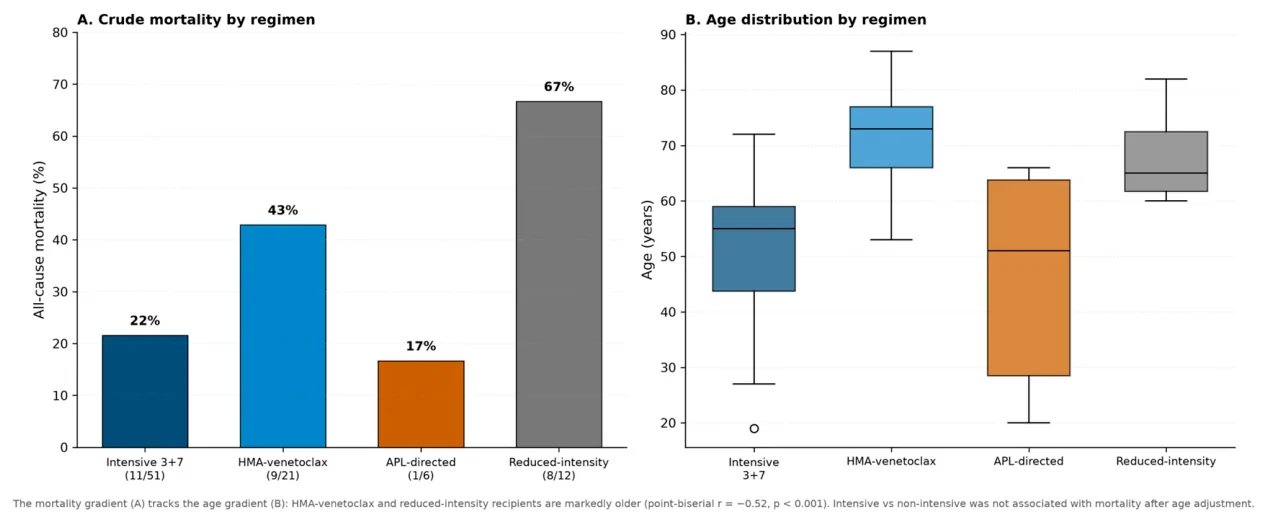
Crude mortality (**A**) and age distribution (**B**) by induction regimen, illustrating confounding by indication (age-intensity point-biserial r = −0.52, *p* < 0.001). Panel (**A**) denominators are patients with known vital status (n = 90): intensive 3+7 n = 51 (one of the 52 patients listed in Table 1 had missing vital status), HMA–venetoclax n = 21, APL-directed n = 6 and reduced-intensity n = 12. Panel (**B**) uses all 91 patients. Four-level regimen contrasts are descriptive only and not intended for between-group inference.

**Regimen and collinearity.** The primary regimen variable was intensive 3+7 versus non-intensive; four-level comparisons (APL-directed n = 6, reduced-intensity n = 12) are descriptive/exploratory only (Supplementary Table S5), with a sensitivity analysis excluding patients with an APL diagnosis. Age–regimen association was quantified by point-biserial correlation and visualised (Figure 2).

**Diagnostics.** Hazard and odds ratios are reported with 95% CIs and the n and event count of every model. Events per variable were low (≈7.3 for the 29-death OS logistic analyses; ≈5.75 for the four-covariate 30-day landmark Cox model); all multivariable estimates are therefore exploratory. No adjustment for multiple comparisons was applied across the several pre-specified model specifications; nominally significant findings are therefore interpreted as exploratory and hypothesis-generating rather than confirmatory. The proportional-hazards assumption was assessed using Schoenfeld residuals and visual log–log plots; no significant violation was detected (global Schoenfeld *p* = 0.40 for the naive Cox model and 0.33 for the 30-day landmark model; Supplementary Table S6), although given the small number of events these diagnostics are exploratory. No imputation was performed because the principal missing variables are structurally absent rather than missing-at-random. Analyses used R version 4.4.3 (R Foundation for Statistical Computing, Vienna, Austria; packages logistf 1.26.1 and survival 3.8.6) with Python version 3.14.3 (Python Software Foundation, Wilmington, DE, USA; pandas 3.0.3, scipy 1.18.0) for data cleaning, descriptive analyses and figure generation; results were cross-checked between the two implementations and both scripts accompany the data.

### 2.7. Ethics

This retrospective study was performed in line with the principles of the Declaration of Helsinki. Approval was granted by the Antalya Training and Research Hospital Ethics Committee, Antalya, Turkey (approval no. 2024-273, decision no. 13/4; approval date: 5 September 2024). The approved protocol covered the retrospective review of the records of patients treated between 2019 and 2025; patients diagnosed after the approval date therefore fall within the approved study period, and all data were abstracted retrospectively from routine clinical records without any study-related intervention. The requirement for informed consent was waived by the ethics committee because of the retrospective design and anonymised data analysis. Full declarations appear in the back matter.

### 2.8. Use of Artificial Intelligence Tools

During the preparation of this work the authors used the large language model assistant Claude Opus 5.5 (Anthropic, San Francisco, CA, USA; https://claude.ai), accessed through Claude Code, for language editing and drafting assistance, for drafting and cross-checking the statistical analysis and figure-generation code (Python and R version 4.4.3), for checking reference formatting, and for document formatting. The tool was not used to generate, fabricate or interpret the underlying clinical data, and no AI-generated material was used as a source. All analyses were executed on the authors’ own dataset, and every output, numerical result, reference and interpretation was reviewed and validated by the authors, who take full responsibility for the content of the manuscript.

## 3. Results

### 3.1. Cohort and Regimen Distribution

The 91 patients had a median age of 59 years (IQR 49–68); 51.6% were male; and 13.2% had secondary or therapy-related AML. Induction was intensive 3+7 in 52 (57.1%), HMA–venetoclax in 21 (23.1%), reduced-intensity in 12 (13.2%) and APL-directed in 6 (6.6%). APL diagnosis and APL-directed regimen were not fully overlapping because regimen grouping was based on the induction protocol documented in treatment records rather than diagnostic category; therefore, APL-related analyses were treated as descriptive, and sensitivity analyses excluded all patients with an APL diagnosis (n = 81 retained; details in Supplementary Table S5). Of the 10 patients with an APL diagnosis, five received all-trans-retinoic-acid-based induction and five received a 7+3-type regimen and were therefore grouped as intensive 3+7; one of the six patients in the APL-directed regimen group carried a non-APL recorded diagnosis. Regimen groups differed sharply in age (median 55, 73, 51 and 65 years respectively; Kruskal–Wallis *p* < 0.001), consistent with allocation of older, less fit patients to lower-intensity backbones [2,12,13] (Table 1, Figure 2B). Median follow-up was 242 days (IQR 84–588); one patient had missing vital status and 25 were not evaluable for CR1.

### 3.2. Infection Burden at Diagnosis and During Induction

**Figure 3:**
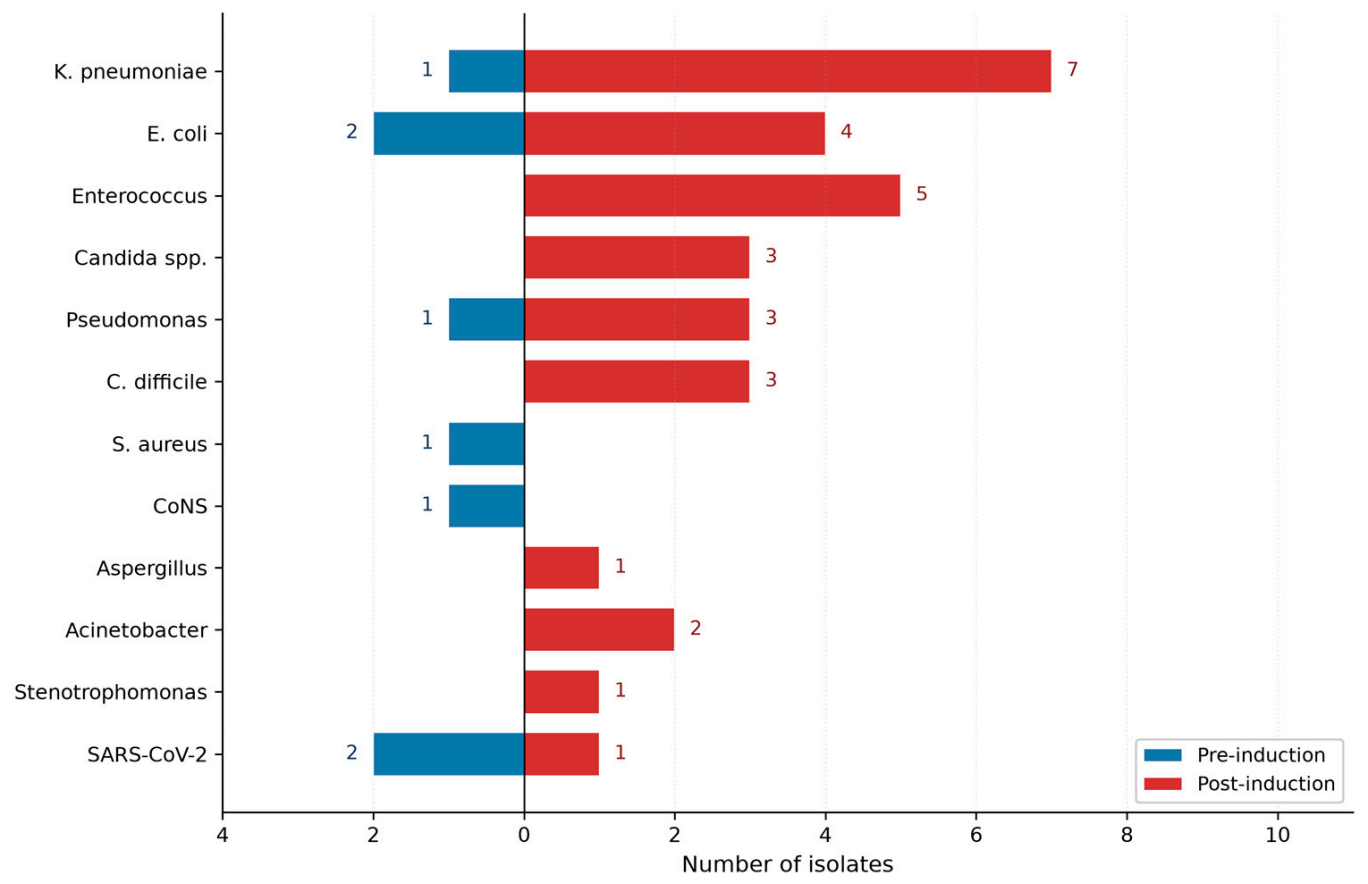
Microbiological spectrum before versus after induction.

Active infection at diagnosis was present in 23/91 patients (25.3%, 95% CI 17.5–35.1). Treatment-emergent infection occurred in 54/91 (59.3%, 95% CI 49.1–68.9) and was predominantly pulmonary (31/91, 34.1%); microbiologically documented infection occurred in 33/91 (36.3%), Gram-negative infection in 13/91 (14.3%), MDR Gram-negative infection in 2/91 (2.2%), bloodstream infection in 2/91 (2.2%), urinary infection in 6/91 (6.6%) and skin/soft-tissue/perianal/catheter infection in 13/91 (14.3%); and breakthrough IFI occurred in 14/91 (15.4%) (Table 2). These frequencies are concordant with contemporary AML induction cohorts [3,14,15]. The microbiological spectrum shifted from a small number of baseline isolates toward a broader post-induction flora dominated by Gram-negative organisms (*Klebsiella pneumoniae*, *Escherichia coli*, *Pseudomonas* spp.) together with the Gram-positive *Enterococcus* spp. (Table 3, Figure 3), mirroring the regional epidemiology of neutropenic bloodstream infection [7,9,10,11].

**Figure 4:**
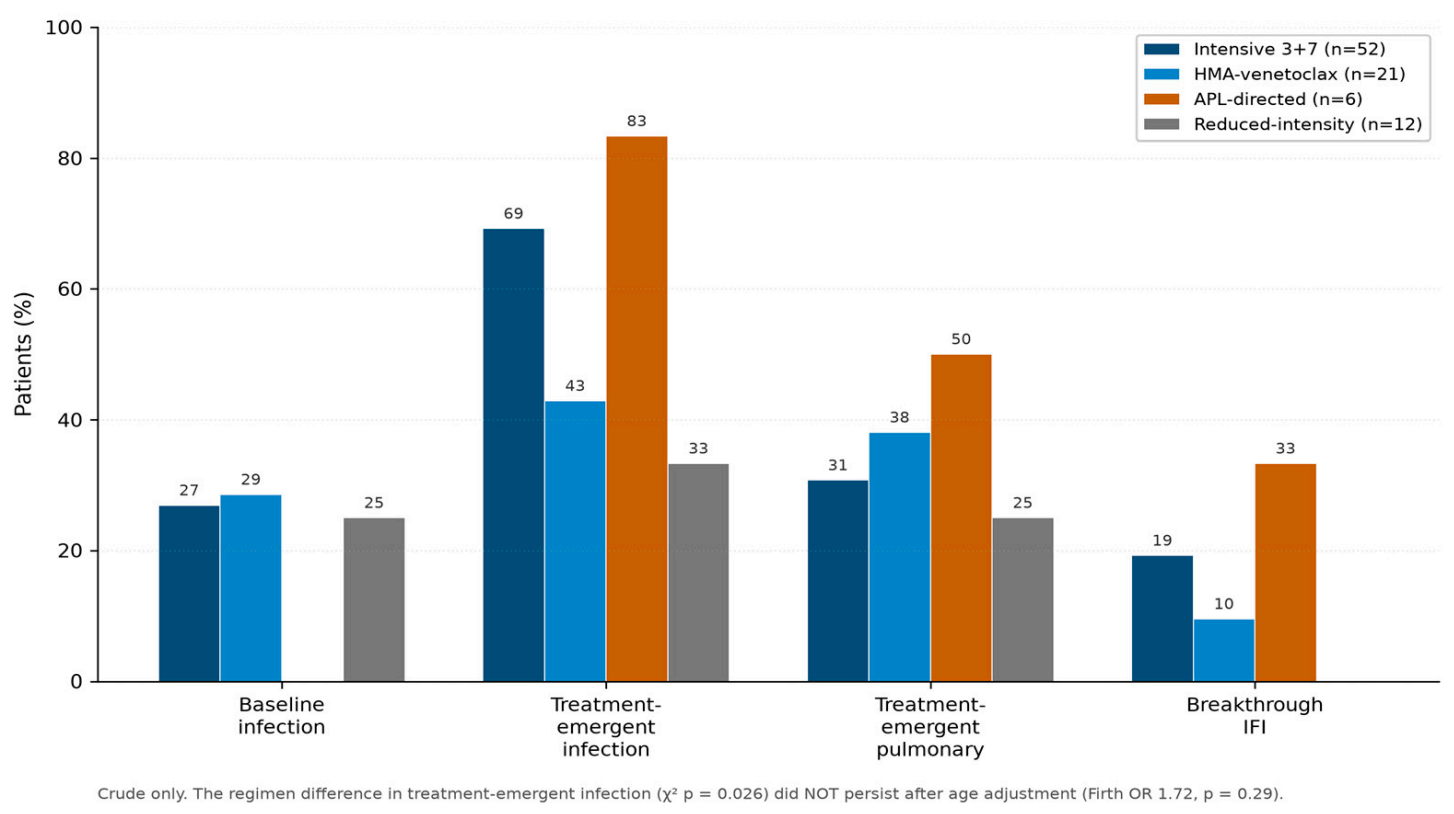
Infection frequency by induction regimen (crude, descriptive). The regimen difference in treatment-emergent infection (χ^2^ *p* = 0.026) did not persist after age adjustment.

Crude treatment-emergent infection frequency differed across regimens (69%, 43%, 83%, 33% for intensive 3+7, HMA–venetoclax, APL-directed and reduced-intensity; χ^2^ *p* = 0.026; Figure 4) but did not persist after adjustment: in Firth logistic regression, intensive 3+7 versus non-intensive was not associated with treatment-emergent infection (OR 1.72, 95% CI 0.63–4.61; *p* = 0.29) once age was included, and baseline infection was likewise unassociated (OR 1.06, 95% CI 0.40–2.87) (Table 4). The crude regimen–infection difference was therefore reduced after adjustment for age rather than reflecting a true effect of the regimen itself; because performance status, comorbidity and frailty were not recorded, the contribution of these unmeasured fitness-related factors could not be assessed directly.

### 3.3. Mortality: Descriptive Rates

All-cause mortality at last follow-up was 29/90 (32.2%, Wilson 95% CI 23.5–42.4). Determinate early mortality was 6/84 (7.1%) at 30 days, 8/82 (9.8%) at 60 days and 12/79 (15.2%) at 90 days (Table 5). Crude all-cause mortality differed markedly by regimen—21.6% (intensive 3+7), 42.9% (HMA–venetoclax), 16.7% (APL-directed) and 66.7% (reduced-intensity) (Figure 2A)—but this gradient closely tracks the age structure of the groups and must not be read as a treatment effect. By infection status, crude mortality was 39.1% with versus 29.9% without baseline infection, and, strikingly, 22.6% with versus 45.9% without treatment-emergent infection (Table 5).

### 3.4. Treatment-Emergent Infection and Mortality: An Immortal-Time-Bias Artefact

**Figure 5:**
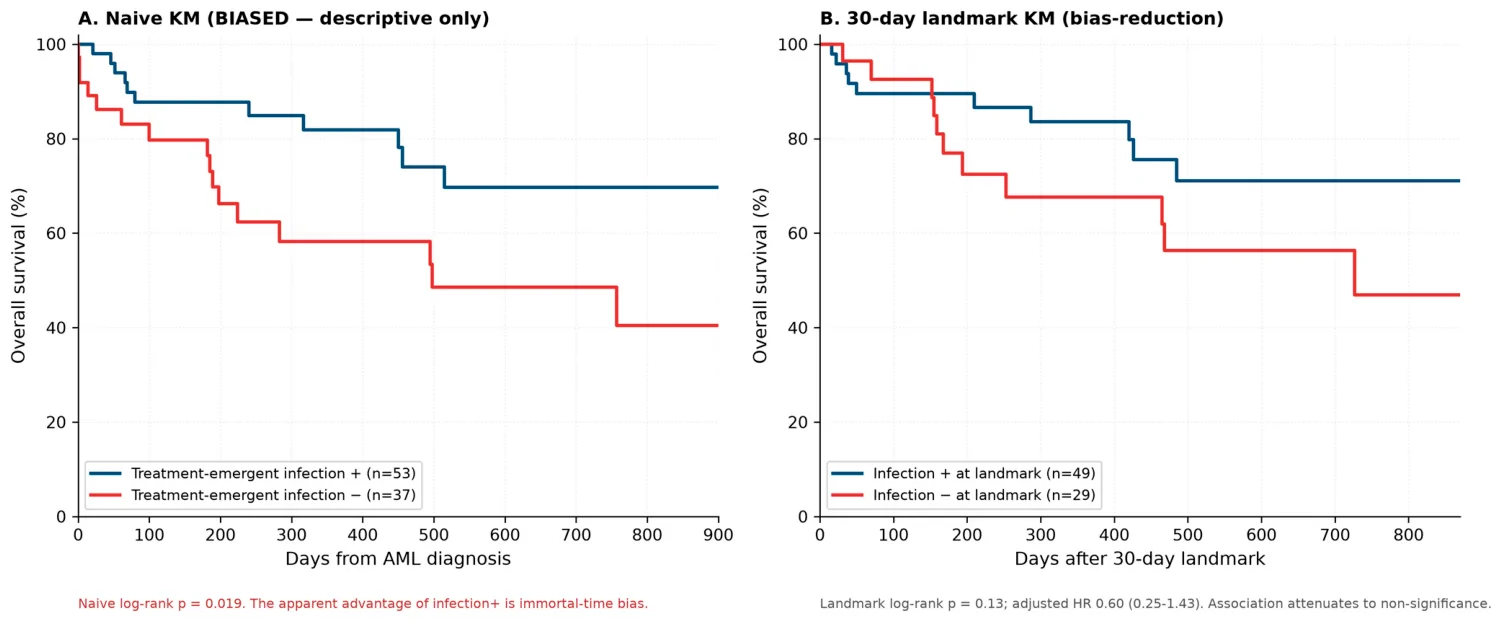
Kaplan–Meier survival by treatment-emergent infection: (**A**) naive (biased, descriptive); (**B**) 30-day landmark (partial bias-reduction). Because exact infection-onset dates were unavailable, landmark infection status reflects whole-induction-course documentation and should be interpreted as partial bias-reduction rather than definitive time-dependent classification. Panel (**A**) includes the 90 patients with known vital status (53 with and 37 without treatment-emergent infection); one of the 54 patients with treatment-emergent infection in the full cohort had missing vital status and is therefore not shown.

**Figure 6:**
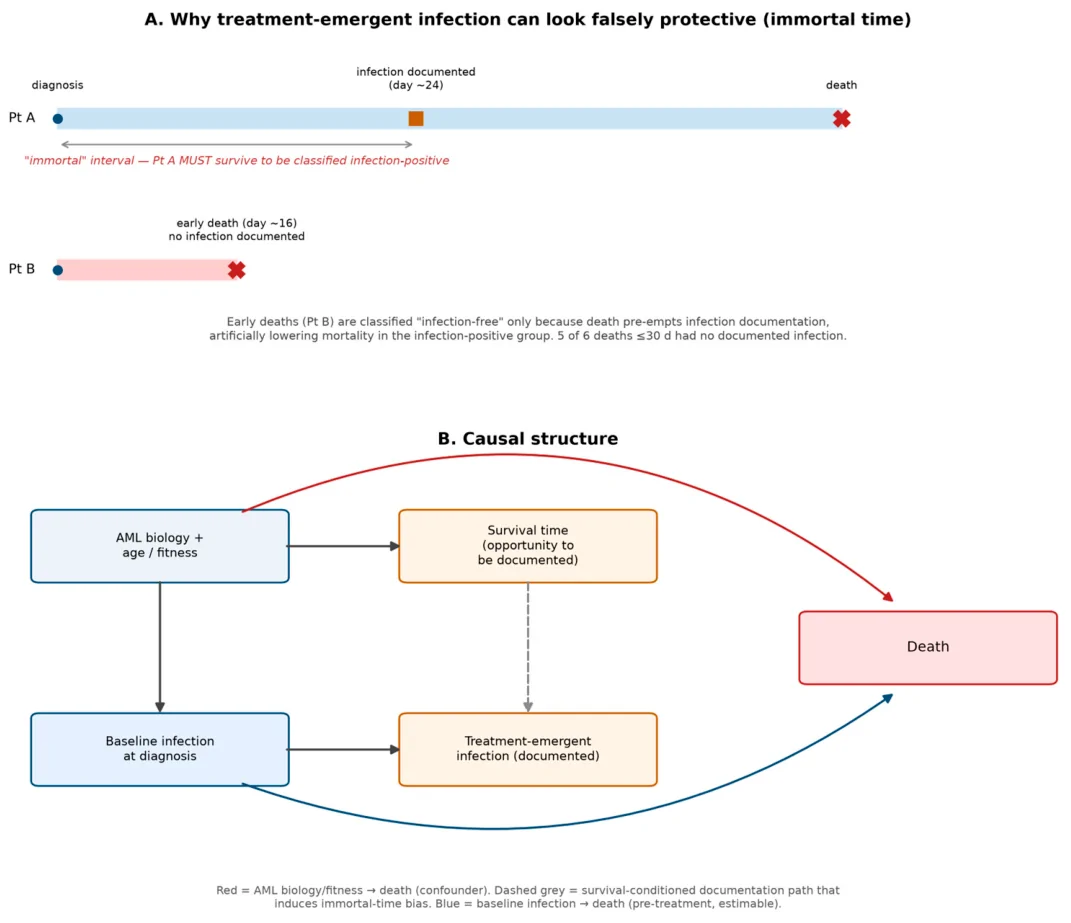
Immortal-time timeline (**A**) and causal diagram (**B**) for treatment-emergent infection. Early deaths are classified infection-free only because death pre-empts infection documentation.

The lower crude mortality among patients with treatment-emergent infection was reproduced in unadjusted time-to-event analysis: median OS was not reached with versus 498 days without treatment-emergent infection (log-rank *p* = 0.019; Figure 5A), and a naive Cox model gave a hazard ratio for treatment-emergent infection of 0.46 (95% CI 0.21–0.99) (Table 6). This biologically implausible “protective” direction is the expected signature of immortal-time bias [18]: a treatment-emergent infection is only ascertainable in a patient who survives to develop it, so the earliest, highest-risk deaths are allocated to the infection-free comparator. The mechanism is explicit in our data: five of the six patients who died within 30 days had no documented treatment-emergent infection (Figure 6).

**Figure 7:**
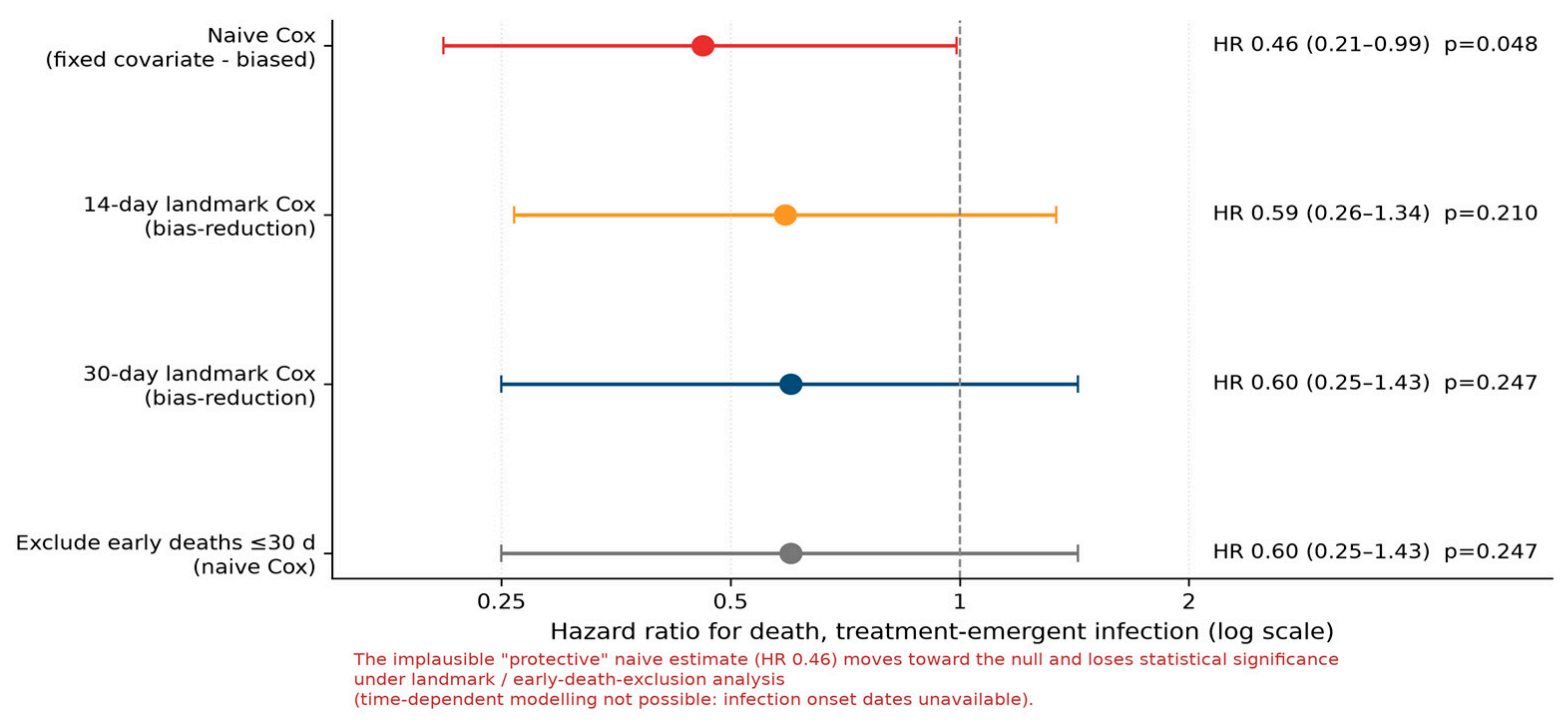
Forest plot of the treatment-emergent infection hazard ratio across naive, 14-day landmark, 30-day landmark and early-death-exclusion models. The naive fixed-exposure Cox model is biased; landmark analyses are a partial bias-reduction. The implausible naive “protective” estimate moves toward the null and loses statistical significance; a true time-dependent model was not estimable because infection-onset dates were unavailable. Numbers analysed and deaths are shown for each model. The dashed vertical line marks a hazard ratio of 1 (no association); horizontal bars show 95% confidence intervals. Colours distinguish the models: red, naive Cox; orange, 14-day landmark; blue, 30-day landmark; grey, exclusion of early deaths.

Three pre-planned bias-aware analyses moved the estimate toward the null and removed its statistical significance (Table 6, Figure 7): the 14-day landmark Cox HR was 0.59 (95% CI 0.26–1.34), the 30-day landmark HR 0.60 (95% CI 0.25–1.43; landmark log-rank *p* = 0.13; Figure 5B), and exclusion of deaths within 30 days gave HR 0.60 (95% CI 0.25–1.43). Sensitivity analyses substituting pulmonary infection (HR 0.66, 95% CI 0.29–1.48) or microbiologically documented infection (HR 0.95, 95% CI 0.41–2.23), and excluding APL patients (HR 0.53, 95% CI 0.24–1.19), were concordant (Supplementary Table S3). Re-anchoring time zero at induction start (n = 84; 26 deaths) gave concordant results—naive HR 0.46 (95% CI 0.20–1.02), 14-day landmark HR 0.53 (95% CI 0.23–1.21) and 30-day landmark HR 0.47 (95% CI 0.19–1.14)—confirming that neither the apparent protective direction nor its loss of significance under landmark analysis is an artefact of measuring survival from diagnosis rather than from treatment start (Supplementary Table S7). The “protective” effect therefore does not survive bias-aware analysis and must not be interpreted as a real survival benefit; equally, because the landmark analyses only partially correct the bias and the confidence intervals are wide, the independent association between treatment-emergent infection and survival could not be reliably estimated in this cohort. We emphasise that, because infection-onset dates were unavailable, even the landmark analyses are a partial bias-reduction rather than a definitive time-dependent correction (Section 4.9).

### 3.5. Baseline Infection, Age and Induction Intensity

In the 30-day landmark Cox model, active infection at diagnosis was associated with higher subsequent mortality (HR 2.56, 95% CI 1.03–6.37), a fragile, exploratory signal that was directionally present but non-significant in the naive model (HR 1.86, 95% CI 0.82–4.21) and the 14-day landmark (HR 2.00, 95% CI 0.83–4.79) and that strengthened as the landmark moved later, consistent with early deaths attenuating it (Table 5). The apparent discordance between this landmark HR and the Firth 30-day mortality model, in which baseline infection had an OR of 0.71 (Table 4), reflects the different outcome windows of the two models rather than a contradiction: the Firth model estimates death within 30 days, whereas the 30-day landmark model, by construction, estimates death after day 30 among 30-day survivors. Early (≤30-day) death was in fact less frequent with baseline infection (1/23 vs. 5/67), whereas later (>30-day) death was more frequent among 30-day survivors (8/21, 38.1%, vs. 15/57, 26.3%; Fisher *p* = 0.40); the landmark signal is therefore driven by deaths beyond the immediate induction period, not by early mortality. This later-death difference was not significant in univariable analysis (HR 1.82, 95% CI 0.76–4.32) and reached nominal significance only after adjustment for age and induction intensity; it was also not significant when time zero was set at induction start (30-day landmark HR 2.18, 95% CI 0.85–5.63; Supplementary Table S7). This estimate rests on only 23 exposed patients and is not adjusted for ELN risk, blood counts or neutropenia duration (all unavailable); it may therefore partly reflect disease severity or frailty rather than an independent effect of infection and should be read as a candidate marker for prospective testing, not a validated predictor. Older age was the most consistent correlate (30-day landmark HR 1.05 per year, 95% CI 1.00–1.10). Intensive versus non-intensive induction was not associated with mortality after adjustment (30-day landmark HR 0.87, 95% CI 0.30–2.54; naive log-rank *p* = 0.089). In Firth logistic models, neither baseline infection nor induction intensity was associated with 30-, 60- or 90-day mortality (Table 4); age–regimen collinearity was strong (point-biserial r = −0.52, *p* < 0.001; Figure 2), so regimen-specific estimates are not interpretable in this sample.

### 3.6. Secondary Outcomes

CR1 was achieved in 51/66 evaluable patients (77.3%); baseline and treatment-emergent infection were statistically independent (Cohen’s κ = 0.01); and allogeneic HSCT was performed in only two patients, reflecting a regional referral pattern and precluding transplant-related analysis.

## 4. Discussion

### 4.1. Principal Findings

In this real-world AML cohort, infection was pervasive: a quarter of patients arrived with active infection at diagnosis and three-fifths developed a treatment-emergent infection, most often pulmonary, frequencies concordant with contemporary induction cohorts [3,14,15]. Three findings are central. First, the crude variation in treatment-emergent infection across induction regimens did not survive adjustment for age, indicating confounding by indication rather than a true effect of the regimen itself. Second, the apparently protective association between treatment-emergent infection and survival is consistent with immortal-time bias and is not supported under bias-aware analysis; because infection-onset dates were unavailable and the landmark analyses only partially correct the bias, its true independent association with survival could not be reliably estimated. Third, after bias-aware analysis it is infection already present at diagnosis, not treatment-emergent infection, that carries a fragile, exploratory association with later mortality.

### 4.2. Treatment-Emergent Infection and Immortal-Time Bias

The naive finding that treatment-emergent infection “protects” against death (HR 0.45) is biologically implausible and methodologically explicable [18]. Because a treatment-emergent infection is only ascertainable in a survivor, the earliest and highest-risk deaths populate the infection-free comparator; our data make this concrete, with five of six early deaths classified infection-free. The pre-planned landmark and early-death-exclusion analyses each move the estimate toward the null (HR ≈ 0.59, with confidence intervals including 1), the concordance of the 14- and 30-day landmarks shows the result is not an artefact of a single landmark choice, and re-anchoring time zero at induction start did not change this picture. This does not establish that treatment-emergent infection is unrelated to survival: because the landmark analyses only partially correct immortal-time and future-information bias and the confidence intervals are wide, the independent association between treatment-emergent infection and survival could not be reliably estimated in this cohort. The principal novelty of this study is therefore not the frequency of infection but the demonstration of why naive fixed-exposure survival models can produce biologically implausible protective effects for treatment-emergent infection, and why time-aware modelling is required. Treatment-emergent infection should be read as a clinical complication and a marker of course, not as an independent determinant of survival.

### 4.3. Baseline Infection as a Candidate Pre-Treatment Supportive-Care Marker

Once immortal-time bias is reduced, infection present at diagnosis emerges as a candidate, though fragile, correlate of later mortality (30-day landmark HR 2.56). This is mechanistically plausible: infection at presentation may index higher disease burden, frailty, delayed or compromised induction, or early mucosal and barrier dysfunction, and disruption of the gut microbiome before and during induction has been shown to predict infectious risk and adverse outcome [25]. Crucially, baseline infection is measurable before the first dose of chemotherapy, which makes it an attractive candidate for risk stratification. It cannot, however, be interpreted as independently prognostic: the estimate is fragile, was nominally significant in only one of several pre-specified specifications, was not significant in univariable analysis or when time zero was set at induction start, rests on 23 exposed patients, and is unadjusted for ELN 2022 risk, baseline counts, neutropenia exposure and fitness, any of which could generate the association through residual confounding. Moreover, no correction for multiple testing was applied across the naive, 14-day landmark, 30-day landmark, induction-anchored and Firth early-mortality models; the single nominally significant p value (0.042) should therefore be read in that multiple-testing context and treated as exploratory and hypothesis-generating rather than confirmatory. It is best regarded as a candidate risk marker requiring prospective validation.

### 4.4. Regimen, Age and Confounding by Indication

Crude mortality ranged from 17% to 67% across regimens, but this gradient mirrors the age structure of the groups (fitness-related factors such as performance status and comorbidity were not recorded and could not be assessed): HMA–venetoclax and reduced-intensity recipients were a median of 10–18 years older than intensive 3+7 recipients (Figure 2) [2,12,13]. After adjustment, intensive versus non-intensive induction was associated with neither infection nor mortality, and age was the dominant correlate, consistent with age and comorbidity as the principal determinants of induction outcome [1,2,12]. The low crude mortality in the small APL-directed group (1/6) should also be read in light of the distinct, predominantly haemorrhagic rather than infective early-death profile of APL [26]. Crude regimen comparisons here therefore cannot support inference about the relative safety of induction strategies, and we make none.

### 4.5. Microbiological and Antifungal Prophylaxis Context

The treatment-emergent predominance of Gram-negative organisms (*K. pneumoniae*, *E. coli*, *Pseudomonas* spp.), alongside the Gram-positive *Enterococcus* spp. (Figure 3), is consistent with the epidemiology of neutropenic bloodstream infection in haematology and with European resistance surveillance, in which Gram-negative bacteraemia and rising ESBL/carbapenem resistance challenge empirical therapy and follow a south-eastern European gradient [7,8,9,10,11]. MDR Gram-negative infection (2.2%) and documented bacteraemia (2.2%) were uncommon here but are almost certainly under-ascertained by retrospective culture records; in endemic regions carbapenem-resistant Gram-negative bacteraemia carries a mortality approaching 50–60% [7,8]. Empirical-therapy strategy should remain guided by local resistance epidemiology and stewardship [5,6,9]. Breakthrough IFI affected 15.2% of patients; mould-active prophylaxis notwithstanding [16,17,27], fungal risk persists within this complex bacterial ecology, and the changing epidemiology of invasive mould disease and emerging azole resistance under widespread prophylaxis are well described [28,29,30,31].

### 4.6. Implications for Supportive-Care Research

From a supportive-care perspective, the key implication is not that treatment-emergent infection is benign, but that its prognostic role cannot be estimated with naive fixed-exposure models. Prospective supportive-care registries in AML should therefore capture exact infection onset, antimicrobial exposure, neutropenia depth and duration, and early death, so that infection can be modelled as a time-dependent exposure and biased inference avoided. If the baseline-infection signal is confirmed in adequately powered, fully adjusted cohorts, active infection at diagnosis (an item already available at the bedside before the first dose of chemotherapy) could plausibly serve as a trigger for intensified supportive care: closer clinical monitoring, earlier infectious-disease consultation, antimicrobial-stewardship review, protocolised culture and imaging pathways, and inclusion in a high-risk supportive-care bundle. We emphasise that these are future research implications, not validated recommendations, and none should change practice before prospective confirmation.

### 4.7. Strengths

Strengths include the joint examination of baseline and treatment-emergent infection in a contemporary, regimen-diverse real-world cohort; reporting of absolute risks alongside model estimates; small-sample-appropriate methods (Firth penalization) [23,24]; an honest pre-specified data-availability audit; formal proportional-hazards diagnostics; and, above all, a pre-planned landmark strategy that exposes rather than propagates immortal-time bias [18,19,20]. Limitations are detailed in Section 4.9.

### 4.8. Future Directions

Definitive evaluation requires a prospective AML infection registry capturing exact infection-onset dates (enabling time-dependent Cox models), neutropenia depth and duration, ELN 2022 genetic risk and molecular data, comorbidity/frailty indices, posaconazole trough levels and venetoclax-azole dose interactions, gut-microbiome sampling, antimicrobial-resistance surveillance, and independent infection adjudication. Such data would allow the baseline-infection signal to be tested as a genuine pre-treatment risk marker rather than a candidate association.

### 4.9. Limitations

This study has important limitations. It is a single-centre retrospective analysis of 91 patients with only 29 deaths, which limits generalisability and yields wide, unstable estimates; subgroups are correspondingly fragile, particularly the APL-directed (n = 6) and reduced-intensity (n = 12) groups, so four-level regimen analyses are exploratory only. Several variables that are central to AML risk and supportive-care interpretation were absent from the source records and could not be analysed: ELN 2022 genetic risk was not derivable (*NPM1* documented in 4/91, *TP53* in 0/91); baseline white-cell, neutrophil and platelet counts, C-reactive protein and procalcitonin were entirely absent (0/91); neutropenia duration, depth, ANC nadir and recovery were not recorded; and ECOG performance status, comorbidity and frailty indices were unavailable. Microbiological infection, especially bloodstream and multidrug-resistant infection, was probably under-ascertained from heterogeneous retrospective culture records, and there was no independent infectious-disease adjudication committee. Most consequentially for the central analysis, exact infection-onset dates were unavailable, so treatment-emergent infection could not be modelled as a true time-dependent covariate; the landmark analyses therefore reduce, but may not fully eliminate, immortal-time and future-information bias, because infection status before each landmark could not be defined with certainty from a whole-induction-course flag. Residual confounding by disease severity, fitness and treatment allocation is expected and cannot be excluded for the baseline-infection signal, and no formal mediation analysis was attempted because the modest sample and the immortal-time structure would render such estimates unstable. Finally, allogeneic transplantation was too infrequent (n = 2) for any transplant-related inference. No correction for multiple testing was applied across the several pre-specified model specifications, and the single nominally significant baseline-infection estimate should be read in that light. These limitations define the requirements for the next generation of AML supportive-care infection registries rather than negating the present methodological observation.

## 5. Conclusions

In newly diagnosed AML managed with universal posaconazole prophylaxis, baseline and treatment-emergent infections were both frequent, and crude infection and mortality rates varied across induction regimens but were substantially reduced after adjustment for age. Treatment-emergent infection appeared protective only in naive fixed-exposure analysis, consistent with immortal-time bias; because landmark analysis only partially corrects this bias, its independent association with survival could not be reliably estimated, and it is best interpreted as a clinical complication and a marker of course rather than a determinant of survival. Baseline infection showed a fragile, exploratory association with later mortality that was nominally significant in only one of several pre-specified specifications, and it cannot be interpreted independently without complete AML-biology, neutropenia and fitness data. Infection-survival analyses in AML induction require time-aware modelling, and the baseline-infection signal merits prospective validation.

## Figures and Tables

**Table 1:** Baseline characteristics by induction regimen (four-level regimen breakdown is descriptive only and not intended for between-group inference).

Variable	Overall	Intensive 3+7	HMA–Venetoclax	APL-Directed	Reduced-Intensity
n	91	52	21	6	12
Age, median (IQR), years	59 (49–68)	55 (44–59)	73 (66–77)	51 (28–64)	65 (62–72)
Age ≥60 years, n (%)	45 (49.5)	11 (21.2)	19 (90.5)	3 (50.0)	12 (100)
Male sex, n (%)	47 (51.6)	27 (51.9)	13 (61.9)	3 (50.0)	4 (33.3)
Diagnosis-to-treatment, days, median (IQR)	3 (2–10)	3 (2–6)	6 (2–19)	2 (0–5)	11 (1–31)
Secondary/therapy-related AML, n (%)	12 (13.2)	3 (5.8)	6 (28.6)	0 (0)	3 (25.0)
APL diagnosis, n (%) ^a^	10 (11.0)	5 (9.6)	0 (0)	5 (83.3)	0 (0)
*FLT3* tested, n ^b^	24	15	5	2	2
*FLT3* positive, n ^b^	11	7	2	1	1
Active infection at diagnosis, n (%)	23 (25.3)	14 (26.9)	6 (28.6)	0 (0)	3 (25.0)
Pre-induction antibiotics, n (%)	28 (30.8)	18 (34.6)	6 (28.6)	1 (16.7)	3 (25.0)

AML: acute myeloid leukaemia, APL: acute promyelocytic leukaemia, HMA: hypomethylating agent, IQR: interquartile range. Kruskal–Wallis *p* < 0.001 for age across regimens. ^a^ APL diagnosis and APL-directed regimen are distinct axes (regimen grouping reflects the documented induction protocol); see Supplementary Table S5. ^b^ *FLT3* tested in 24/91; reported descriptively only and not used as a model covariate.

**Table 2:** Infection burden at diagnosis and during induction.

Endpoint	n/N	%	95% CI ^a^
Active infection at diagnosis	23/91	25.3	17.5–35.1
Treatment-emergent infection	54/91	59.3	49.1–68.9
Pulmonary infection	31/91	34.1	25.2–44.3
Bloodstream infection	2/91	2.2	0.6–7.7
Urinary tract infection	6/91	6.6	3.1–13.6
Skin/soft-tissue/perianal/catheter infection	13/91	14.3	8.5–22.9
Microbiologically documented infection	33/91	36.3	27.1–46.5
Gram-negative infection	13/91	14.3	8.5–22.9
MDR Gram-negative infection	2/91	2.2	0.6–7.7
Breakthrough invasive fungal infection (EORTC/MSGERC 2020)	14/91	15.4	9.4–24.2

MDR: multidrug-resistant, EORTC/MSGERC: European Organisation for Research and Treatment of Cancer/Mycoses Study Group Education and Research Consortium. ^a^ Wilson 95% confidence interval.

**Table 3:** Microbiological spectrum before and after induction.

Organism	Pre-Induction	Post-Induction	Total
*Klebsiella pneumoniae*	1	7	8
*Escherichia coli*	2	4	6
*Enterococcus* spp.	0	5	5
*Pseudomonas* spp.	1	3	4
*Candida* spp.	0	3	3
*Clostridioides difficile*	0	3	3
*Acinetobacter* spp.	0	2	2
*Aspergillus* spp.	0	1	1
*Stenotrophomonas* spp.	0	1	1
*Staphylococcus aureus*	1	0	1
Coagulase-negative staphylococci	1	0	1
SARS-CoV-2	2	1	3

Counts are documented isolates; “pre-induction” and “post-induction” denote the timing of sampling relative to induction start.

**Table 4:** Firth penalised logistic regression models for early mortality and treatment-emergent infection.

Outcome (n; Events)	Term	OR (95% CI) ^a^	*p*
30-day mortality (n = 84; 6)	Age (per year)	1.00 (0.95–1.08)	0.988
	Baseline infection	0.71 (0.07–3.79)	0.716
	Intensive 3+7	0.81 (0.14–5.54)	0.821
60-day mortality (n = 82; 8)	Age (per year)	0.99 (0.95–1.05)	0.777
	Baseline infection	1.01 (0.18–4.28)	0.988
	Intensive 3+7	0.72 (0.15–3.63)	0.674
90-day mortality (n = 79; 12)	Age (per year)	1.02 (0.97–1.09)	0.401
	Baseline infection	0.89 (0.21–3.14)	0.863
	Intensive 3+7	1.52 (0.38–7.05)	0.568
Treatment-emergent infection (n = 91; 54)	Age (per year)	0.97 (0.94–1.01)	0.129
	Baseline infection	1.06 (0.40–2.87)	0.906
	Intensive 3+7	1.72 (0.63–4.61)	0.285

OR odds ratio. ^a^ Firth penalised logistic regression (R package logistf); profile-penalised-likelihood confidence intervals.

**Table 5:** Mortality and supportive-care outcomes by baseline infection and induction regimen (regimen rows are descriptive only and not intended for between-group inference).

Stratum	n	30-Day ^a^	60-Day ^a^	90-Day ^a^	All-Cause (Last Follow-Up)	CR1	Allo-HSCT
Overall	90	6/84 (7.1)	8/82 (9.8)	12/79 (15.2)	29/90 (32.2)	51/66	2
Baseline infection, present	23	1/22 (4.5)	2/22 (9.1)	3/22 (13.6)	9/23 (39.1)	15/18	0
Baseline infection, absent	67	5/62 (8.1)	6/60 (10.0)	9/57 (15.8)	20/67 (29.9)	36/48	2
Intensive 3+7	51	3/46 (6.5)	4/45 (8.9)	7/44 (15.9)	11/51 (21.6)	35/40	2
HMA–venetoclax	21	1/21 (4.8)	1/20 (5.0)	2/18 (11.1)	9/21 (42.9)	8/13	0
APL-directed	6	1/6 (16.7)	1/6 (16.7)	1/6 (16.7)	1/6 (16.7)	4/4	0
Reduced-intensity	12	1/11 (9.1)	2/11 (18.2)	2/11 (18.2)	8/12 (66.7)	4/9	0

Values are n/N (%) unless stated. CR1: first complete remission, Allo-HSCT: allogeneic haematopoietic stem-cell transplantation. ^a^ 30/60/90-day denominators are determinate subsets (patients alive but censored before the cut-off were excluded; Supplementary Table S4). Regimen contrasts are confounded by age; the treatment-emergent-infection contrast is distorted by immortal-time bias (Table 6).

**Table 6:** Bias-aware survival models for treatment-emergent infection as the exposure of interest.

Model	HR (95% CI) ^a^	*p*	n	Deaths	Interpretation
Naive Cox (fixed baseline covariate)	0.46 (0.21–0.99)	0.048	90	29	Biased; immortal time likely
14-day landmark Cox	0.59 (0.26–1.34)	0.210	84	25	Bias-reduction (partial)
30-day landmark Cox	0.60 (0.25–1.43)	0.247	78	23	Bias-reduction (partial)
Excluding early deaths ≤30 days	0.60 (0.25–1.43)	0.247	84	23	Sensitivity
Time-dependent Cox	NA	NA	NA	NA	Not estimable; infection-onset dates unavailable

HR: hazard ratio, CI: confidence interval. ^a^ Co-covariates in every fitted model: age, intensive 3+7, baseline infection. In the naive model: age HR 1.04 (1.00–1.08), intensive 0.92 (0.36–2.34), baseline infection 1.86 (0.82–4.21). In the 30-day landmark model: age HR 1.05 (1.00–1.10, *p* = 0.031), intensive 0.87 (0.30–2.54), baseline infection 2.56 (1.03–6.37, *p* = 0.042). In landmark models treatment-emergent infection reflects whole-induction-course documentation, not a dated event. The naive→landmark attenuation of the treatment-emergent-infection HR (0.46→0.60, losing significance) is the central methodological result; the proportional-hazards assumption was not violated (Supplementary Table S6).

## Data Availability

De-identified data and analysis code are available from the corresponding author upon reasonable request, subject to institutional approval and Turkish data-protection regulations.

## Supplementary Materials

The following supporting information can be downloaded at https://www.mdpi.com/article/10.3390/jcm15207841/s1: Table S1: Variable dictionary and data availability; Table S2: Operational definitions of infectious endpoints and microbiological categories; Table S3: Sensitivity analyses (naive Cox models; exposure as labelled); Table S4: Patients indeterminate for early-mortality endpoints, by infection and induction-intensity group; Table S5: Four-level regimen descriptive outcomes, and clarification of APL diagnosis versus APL-directed regimen; Table S6: Schoenfeld proportional-hazards diagnostics; Table S7: Sensitivity analyses added at revision: time zero at induction start, and early versus later death by baseline infection.

## Abbreviations

AML, acute myeloid leukaemia; ANC, absolute neutrophil count; APL, acute promyelocytic leukaemia; CI, confidence interval; CR1, first complete remission; ELN, European LeukemiaNet; ESBL, extended-spectrum β-lactamase; HMA, hypomethylating agent; HR, hazard ratio; HSCT, haematopoietic stem-cell transplantation; IFI, invasive fungal infection; IQR, interquartile range; MDR, multidrug-resistant; OR, odds ratio; OS, overall survival; STROBE, Strengthening the Reporting of Observational Studies in Epidemiology.
