# Supplementary material for: Infectious Burden and Immortal-Time Bias During AML Induction: A Supportive-Care Cohort Under Posaconazole Prophylaxis
Source: J Clin Med. 2026 Oct 11;15(20):7841. doi: 10.3390/jcm15207841 (PMC13705091; doi:10.3390/jcm15207841)
Supplement: Supplementary file 1 [file jcm-15-07841-s001.zip › jcm-4559315-supplementary.pdf]

Supplementary Materials

Article: Infectious Burden and Immortal-Time Bias During AML Induction: A Supportive-Care Cohort Under Posaconazole Prophylaxis

Journal: *Journal of Clinical Medicine*

Authors: Hilmi Erdem Gözden, Halime Betül Tokat, Hamide Tuğba Uluçay, Fatma Tuğçe Bayrak, Recep Kaya, Alperen Burak Kocabıyık, Süleyman Emre Okutan, İbrahim Ethem Pınar, Erdal Kurtoğlu, Volkan Karakuş

Corresponding author: Hilmi Erdem Gözden, Department of Hematology, Bursa Yüksek İhtisas Training and Research Hospital, University of Health Sciences, Bursa, Turkey. E-mail: erdemgozden83@gmail.com

Supplementary Table S1. Variable dictionary and data availability

At revision, one patient diagnosed in December 2015, before the start of the ethics-approved study period (2019-2025), was excluded by protocol rule; all supplementary tables refer to the resulting n = 91 analytic cohort (90 with known vital status).

Variable	Availability	Detail
Age	Available	91/91
Sex	Available	91/91
Induction regimen	Available	91/91
AML subtype (de novo / secondary / therapy-related)	Available	90/91
Diagnosis-to-treatment interval	Available	87/91
Follow-up time / death status	Available	90/91 (1 missing vital status)
30/60/90-day mortality	Partial (computed)	determinate 84/82/79; 6/8/11 indeterminate (alive, censored before cut-off)
FLT3 status	Partial	24/91 tested (11 positive)
NPM1	Partial	4/91
APL t(15;17)	Partial	10/91
Baseline WBC / ANC /	Absent	0/91 (empty fields)

Variable	Availability	Detail
platelets / haemoglobin / LDH / CRP / procalcitonin		
ELN 2022 genetic-risk category	Absent	not derivable (NPM1 4/91, TP53 0/91)
ECOG / Charlson / HCT-CI / frailty index	Absent	no field
Neutropenia duration/depth, ANC nadir, recovery	Absent	no field
Infection-onset dates	Absent	no field (precludes time-dependent Cox)
Posaconazole trough / venetoclax dose	Absent	no field

Supplementary Table S2. Operational definitions of infectious endpoints and microbiological categories

Endpoint	Operational definition
Active infection at diagnosis	Clinically identified infectious focus and/or documented infection present before induction start. Isolated fever without a clinical, radiological or microbiological focus was not classified as active infection unless the treating team documented and treated it as a clinical infection.
Treatment-emergent infection	New clinically, microbiologically and/or radiologically documented infection after induction start. Febrile neutropenia alone was not sufficient unless accompanied by a documented clinical focus, microbiological evidence or radiological evidence.
Clinically documented infection	Clinical/radiological focus without microbiological confirmation.
Microbiologically documented infection	Clinically meaningful pathogen from blood, urine, respiratory, catheter, wound, stool or sterile-site culture. Isolated coagulase-negative staphylococci treated as contaminants unless recovered from multiple sets with

Endpoint	Operational definition
	a compatible syndrome; *Candida* from non-sterile sites and asymptomatic candiduria not counted as invasive infection.
Bloodstream infection	Positive blood culture with a compatible systemic syndrome (same skin-contaminant rule).
Pulmonary infection	Compatible clinical syndrome supported by chest imaging (computed tomography where available, otherwise chest radiography). Pulmonary events meeting EORTC/MSGERC criteria for possible/probable fungal pneumonia were additionally counted under breakthrough IFI; this overlap is noted in the main text.
Urinary tract infection	Compatible symptoms and/or systemic signs with a positive urine culture.
Skin/soft-tissue/perianal/catheter infection	Clinically documented localised infection at the respective site.
Clostridioides difficile infection	Diarrhoea with a positive toxin/PCR assay.
MDR Gram-negative infection	Resistance defined per standard international categories (extended-spectrum β-lactamase [ESBL]/third-generation-cephalosporin-resistant; carbapenem-resistant).
Breakthrough invasive fungal infection (IFI)	EORTC/MSGERC 2020 proven/probable/possible; reported here as a descriptive frequency among the infection endpoints.

Infection adjudication was by treating-records review; no independent infectious-disease adjudication committee was convened.

Supplementary Table S3. Sensitivity analyses (naive Cox models; exposure as labelled)

Analysis	Exposure	HR (95% CI)	p	n	Deaths
Full cohort	Treatment-emergent	0.46 (0.21-0.99)	0.048	90	29

Analysis	Exposure	HR (95% CI)	p	n	Deaths
	infection				
Excluding APL (by diagnosis)	Treatment-emergent infection	0.53 (0.24-1.19)	0.125	80	28
Excluding early deaths ≤30 days	Treatment-emergent infection	0.60 (0.25-1.43)	0.247	84	23
Pulmonary infection as exposure	Pulmonary infection	0.66 (0.29-1.48)	0.309	90	29
Microbiologically documented infection as exposure	Microbiologically documented infection	0.95 (0.41-2.23)	0.914	90	29

All models adjusted for age, intensive 3+7 and baseline infection. HR hazard ratio, CI confidence interval.

Supplementary Table S4. Patients indeterminate for early-mortality endpoints, by infection and induction-intensity group

Cut-off	Indeterminate, n	Baseline infection +	Baseline infection -	Treatment-emergent +	Treatment-emergent -	Intensive 3+7	Non-intensive	Determinate denominator
30-day	6	1	5	3	3	5	1	84
60-day	8	1	7	4	4	6	2	82
90-day	11	1	10	5	6	7	4	79

Indeterminate = alive at last documented contact, but that contact occurred before the cut-off (incomplete follow-up records or transfers). Exclusions were balanced across treatment-emergent infection status (Fisher p = 0.69 at day 30).

Supplementary Table S5. Four-level regimen descriptive outcomes, and clarification of APL diagnosis versus APL-directed regimen

Regimen	n	Median age	30-day	60-day	90-day	All-cause	Treatment-emergent infection	Breakthrough IFI
Intensive 3+7	51	55	3/46 (7%)	4/45 (9%)	7/44 (16%)	11/51 (22%)	36/52	10/52
HMA-venetoclax	21	73	1/21 (5%)	1/20 (5%)	2/18 (11%)	9/21 (43%)	9/21	2/21
APL-directed	6	51	1/6 (17%)	1/6 (17%)	1/6 (17%)	1/6 (17%)	5/6	2/6
Reduced-intensity	12	65	1/11 (9%)	2/11 (18%)	2/11 (18%)	8/12 (67%)	4/12	0/12

APL classification clarification. Ten of 91 patients (11.0%) had an acute promyelocytic leukaemia (APL) diagnosis, whereas 6/91 (6.6%) received an APL-directed (all-trans-retinoic-acid-based) induction regimen. Of the 10 APL-diagnosis patients, 5 received ATRA-based induction and 5 received a 7+3-type regimen (classified as intensive 3+7); 1 of the 6 APL-directed-regimen patients carried a non-APL recorded diagnosis. Regimen grouping reflects the documented induction protocol rather than the diagnostic category. Sensitivity analyses excluded all 10 patients with an APL diagnosis (n = 81; 80 vital-status-evaluable; 28 deaths).

Supplementary Table S6. Schoenfeld proportional-hazards diagnostics

Model	Term	Schoenfeld p
Naive Cox	Age	0.164
Naive Cox	Intensive 3+7	0.103
Naive Cox	Baseline infection	0.627
Naive Cox	Treatment-emergent infection	0.937
Naive Cox	GLOBAL	0.400
30-day landmark Cox	Age	0.338

Model	Term	Schoenfeld p
30-day landmark Cox	Intensive 3+7	0.081
30-day landmark Cox	Baseline infection	0.637
30-day landmark Cox	Treatment-emergent infection	0.200
30-day landmark Cox	GLOBAL	0.331

No statistically significant violation of the proportional-hazards assumption was detected; given the small number of events these diagnostics are exploratory.

Supplementary Table S7. Sensitivity analyses added at revision: time zero at induction start, and early versus later death by baseline infection

(A) Cox models with time zero at induction start. Follow-up was defined as time from diagnosis minus the recorded diagnosis-to-treatment interval (available for 86 of 90 patients with known vital status); two patients without follow-up beyond induction start were excluded (n = 84; 26 deaths). Landmarks were applied from induction start. All models adjusted for age, intensive 3+7 and baseline infection.

Model (time zero = induction start)	n	Deaths	Treatment-emergent infection HR (95% CI), p	Baseline infection HR (95% CI), p	Age HR (95% CI), p
Naive Cox	84	26	0.46 (0.20-1.02), 0.055	2.02 (0.88-4.66), 0.098	1.04 (1.00-1.09), 0.056
14-day landmark Cox	81	24	0.53 (0.23-1.21), 0.131	1.96 (0.81-4.70), 0.134	1.04 (0.99-1.08), 0.117
30-day landmark Cox	74	21	0.47 (0.19-1.14), 0.096	2.18 (0.85-5.63), 0.106	1.06 (1.00-1.11), 0.035
Reference: naive Cox, time zero = diagnosis, same 84 patients	84	26	0.44 (0.20-0.99), 0.048	-	-

Compared with the diagnosis-anchored models in Table 6, the direction of the treatment-emergent-infection estimate and its loss of statistical significance under

landmark analysis were unchanged. The baseline-infection estimate was not statistically significant in any induction-anchored model.

(B) Early (≤30-day) versus later (>30-day) death by baseline infection (n = 90). This reconciles the Firth 30-day mortality model (baseline infection OR 0.71, Table 4), which estimates death within 30 days, with the 30-day landmark Cox model (HR 2.56, Table 6), which estimates death after day 30 among 30-day survivors.

Baseline infection	n	Deaths ≤30 days	Alive beyond 30 days	Deaths >30 days among 30-day survivors
Present	23	1 (4.3%)	21	8/21 (38.1%)
Absent	67	5 (7.5%)	57	15/57 (26.3%)

Later death, present vs absent: Fisher exact p = 0.40; univariable 30-day landmark Cox HR 1.82 (95% CI 0.76-4.32), p = 0.18. The adjusted 30-day landmark HR of 2.56 (Table 6) reached nominal significance (p = 0.042) only after adjustment for age and induction intensity, and no correction for multiple testing was applied.
